# Some Nervous Complications Occurring in Pulmonary Tuberculosis

**Published:** 1905-09-30

**Authors:** Isabella Mears

**Affiliations:** Medical Superintendent, Woodburn Sanatorium, Edinburgh


### Hospital Clinics.

### SOME NERVOUS COMPLICATIONS OCCURRING IN PULMONARY TUBERCULOSIS.

By ISABELLA MEARS, L.R.C.P.I., Medical Superintendent, Woodburn Sanatorium, Edinburgh.

IN all recent writings on the subject of open-air or rather of sanatorium treatment of pulmonary tuberculosis, there seems to be a universal opinion that early cases are comparatively easy to cure. It is conceded by all, whether or not they approve of sanatoria for the treatment of consumptives, that nowadays a patient is not certainly doomed to an early grave because physical signs indicate the presence of tuberculosis in the lungs. It has been proved, beyond dispute, that such a patient stands a very good chance of recovery, and of a consequent long and healthy life, if only he has it in his power to follow out certain rules, and to give sufficient time for the purpose of killing out the bacillus and of regaining his normal physical strength. At the same time it is frequently and earnestly urged by those who see no good in sanatorium treatment, and it cannot fail to be granted by those who are in favour of it, that even in apparently simple cases delay in the progress towards complete recovery, and disappointment as to the ultimate good result, have too frequently occurred. Certain factors militate against progress. Certain types of the disease are persistent and seem to be incurable.

Just to know wherein a difficulty lies, is often a great step towards victory. Therefore if we can ascertain what factors have led to delay and failure in the past, we may learn how to guard against their occurrence, and how to overcome them when they do occur. This knowledge can only be attained by careful study of the records of a number of cases treated during a period of several years, and by allowing sufficient time for observation of their after history.

The observations based on the records of all cases admitted to Woodburn Sanatorium during a period of four years (1899 to 1903) show that some complications, though apparently of little account, are yet very serious because they may lead to a fatal issue. For instance, it has occurred that a patient who, during residence, had comparatively little lung disease, but who could not be made to understand the importance of his own individual effort, has in the long run done badly. While, on the other hand, some complications, especially some tuberculous complications, formerly considered surely fatal, have been entirely overcome by steady and careful treatment.

For practical purposes, the complications occurring in the course of pulmonary tuberculosis may be considered as functional and organic. Functional complications are often simply the exaggeration of one or more of the ordinary symptoms of the disease, but they are liable to become organic when they are not checked by effective treatment. It is therefore important that thorough measures should be adopted for the treatment of cases in the earliest stages of tuberculosis, before the aggravation of the functional symptoms which later on become difficult complications. It is obvious that the length of time required for the removal of these secondary organic changes must have an important influence on the prognosis.

Organic complications are sometimes purely tubercular in their origin. When they are non-tubercular, they are generally due to a marked devitalisation of the tissues, which is probably the result of slow and continuous poisoning by a toxin secreted by, or connected with the growth and increase of the tubercle bacillus. This devitalisation affects all the tissues, but especially the nervous and muscular systems, and through them various important organs by imperfect innervation and by wasting of substance.

In the present paper only those symptoms will be referred to which may be classed definitely as nervous. These can be observed in greater or lesser degree in the majority of cases which come under treatment. They are sometimes to be accounted for by a slight deficiency of inhibition of the emotions or of the judgment. Patients thus affected are easily moved to laughter or to tears. They cannot be led to see the importance of "keeping the rules" strictly. They depend too much on the will that guides them. They do not know how to lend their own strength of will to the accomplishment of the cure. This lack of appreciation of the importance of individual effort is, apparently, at the moment a small matter, but it is far-reaching in its effects. If the physician cannot by his influence and teaching overcome it and infuse in its place a hopeful, steady, and courageous impulse, it will almost certainly lead, sooner or later, to a fatal issue.

In certain cases the nervous symptoms show themselves in a deliberate wish to evade the means used for cure, and this seems to indicate a perversion of the judgment. In such a case it is a difficult matter to inspire the enthusiasm that is required to bring about a successful issue. Let the focus of disease be ever so small, the ultimate prognosis must still be grave.

A deficiency of nerve force occurs in cases in which the original cause of the breakdown is overwork or previous mental disease. The patient is really anxious to get well, but cannot bring his force of will to the point of taking sufficient nourishment to combat the disease. The only effective treatment lies in securing complete mental rest, and in arranging that all food given shall be most nutritious. Even so, and under the best conditions, it may be expected that the cure will be slow.

Insomnia frequently results from overwork or nervous overstrain. This is a grave symptom which must be relieved before much progress can be made. When a patient is admitted in an exhausted condition, being sleepless and unable to take much solid nourishment, it is our practice to begin the dietetic treatment by the administration of raw beef-juice, giving the extract of a pound of fresh beef three times a day. It has been found experimentally that this diet has a marvellously soporific effect, and that by its means the nervous system is quickly soothed. The latter good result is partly due to the sleep obtained, partly also to the stimulant and nutritive action of the beef. The improvement in the blood in such cases is often marked by an access of 12 per cent. or 13 per cent. of hæmoglobin in the course of a week. It is interesting to note that acute influenza has been quickly cured when treated in the same manner. In the case of a lady who was subject to influenzal attacks, and who asserted that it was always six months before she regained her tone, the effect of the raw beef-juice treatment was that she recovered the lost strength and weight in the short space of 14 days.

There is a very general belief that the prevailing attitude of mind in pulmonary tuberculosis is one of hope, so that one hears of patients planning for a trip abroad when they are in reality within a few hours of death. The experience in the years of work here has been that, on the contrary, there is frequently a marked despondency, which only yields to hope and cheerfulness as recovery becomes assured. This depression has at times been so pronounced as to interfere with the cure, being then, probably, a true melancholia.

When the illness is protracted it happens frequently that the patient becomes self-centred—a condition which is accentuated as weakness is more marked. The danger of self-absorption is one which must be guarded against in all chronic diseases. For this reason it is well that patients should be encouraged to take up some work or course of study as soon as they are fit for it. Some patients who have in the long run done extremely well date their best progress from the time when they were allowed to resume a part of their usual occupation. It is essential, however, that the work for a year or so should not involve any severe mental or physical strain, also that ample provision be made for resting after meals, for abundant wholesome food, and for continuous fresh air.

All these phases or symptoms point to a general loss of power in the nervous system, or to an obscure change in function, as when the temperature swings violently up or down on the least excitement or exertion.

The nerve-disturbance is sometimes localised, as neuritis, to a certain anatomical distribution. For instance, neuralgic pain occurs from no other ascertainable cause than an undue irritability of the sensory nerves. Continual restless movements of the limbs or jerky movements of various muscles occur, showing a want of power of control in the motor nerves. There is often flushing, perspiration, easily-quickened pulse-rate, palpitation—due, probably, to want of tone in the nervous mechanisms controlling the circulation. There is also almost constantly a loss of staying power, the patient not being able to stand any anxiety or mental strain without serious risk of a relapse. The various nervous symptoms enumerated above do not indicate that any tubercular involvement of the brain or spinal cord has taken place, but simply that the nervous system is devitalised. The indications for treatment are, therefore, complete mental rest to begin with and some interesting occupation as soon as it can be safely taken up. Particular attention must also be given to the dietary, which requires to be easily digestible as well as very nutritious. Raw or underdone beef has been found to be particularly valuable. Occasionally some simple sedative is helpful, and small doses of strychnine and arsenic have been administered with benefit. Above all the personal influence of physician and nurse has more beneficial effect than many drugs in the cure of nervous complications.

